# Rupture spontanée d’un kyste hydatique du foie dans la cavité péritonéale avec une membrane proligère intacte: à propos d’un cas et revue de la littérature

**DOI:** 10.11604/pamj.2018.30.174.15054

**Published:** 2018-06-26

**Authors:** Baccouch Seifeddine, Challakhi Amel, Talbi Ghofrane, Bacha Dhouha, Gharbi Lassaad, Bayar Rached, Khalfallah Mohamed Taher

**Affiliations:** 1 Service de Chirurgie Viscérale CHU Mongi Slim, Sidi Daoued La Marsa, Tunisie; 2 Service d’Anatomopathologie et de Cytogénétique CHU Mongi Slim, Sidi Daoued La Marsa, Tunisie

**Keywords:** Kyste hydatique, foie, rupture, membrane proligère intacte, péri kyste, chirurgie, Tunisie, Hydatid cyst, liver, rupture, intact proligerous membrane, pericyst, surgery, Tunisia

## Abstract

La rupture intrapéritonéale du kyste hydatique du foie avec une membrane proligère intacte est rare. Nous rapportons le cas d'une patiente âgée de 63 ans qui avait consulté pour douleur de l'hypochondre droit sans fièvre ni ictère. L'imagerie trouvait un kyste hydatique du segment IV du foie associé à une lésion kystique pelvienne de 12cm x 06cm. L'exploration chirurgicale trouvait un kyste hydatique univésiculaire du foie rompu avec une membrane proligère intacte et libre dans la cavité péritonéale. Le but de ce travail est de rapporter un nouveau cas de rupture intrapéritonéale d'un kyste hydatique du foie avec membrane proligère intacte et de préciser les difficultés diagnostiques et thérapeutiques rencontrées au décours de cette complication.

## Abstract (en)

Rupture of hydatid cyst of liver with intact proligerous membrane is rare. We report the case of a 63 year-old female patient presenting with right hypocondrium pain without fever or jaundice. Diagnostic imaging examination showed hydatid cyst in segment IV of liver associated with pelvic cystic lesion measuring 12 cm x 06 cm. Surgical exploration revealed ruptured unilocular hydatid cyst of the liver with intact proligerous membrane and free in the peritoneal cavity. This study aims to report a new case of intraperitoneal rupture of hydatid cyst of liver with intact proligerous membrane and to highlight the diagnostic and therapeutic difficulties due to this complication.

## Introduction

La rupture intrapéritonéale du kyste hydatique du foie est rare (1 à 2%) [1,2]. Elle expose à deux risques, à savoir l'hydatidose péritonéale et l'anaphylaxie aigue. La rupture intrapéritonéale du kyste hydatique du foie avec une membrane proligère intacte est une forme particulière. Seulement cinq cas sont rapportés [3-7]. L'intégrité de la membrane proligère protège contre les complications potentielles à condition qu'un traitement adéquat soit réalisé en urgence. Le but de ce travail est de rapporter un nouveau cas de rupture intrapéritonéale d'un kyste hydatique du foie avec membrane proligère intacte et de préciser les difficultés diagnostiques et thérapeutiques rencontrées au décours de cette complication.

## Patient et observation

**Figure 1:**
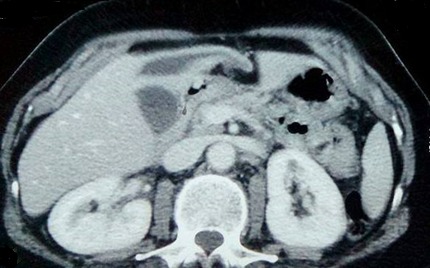
Une coupe scannographique montrant un kyste hydatique du segment IV du foie affaissé

**Figure 2:**
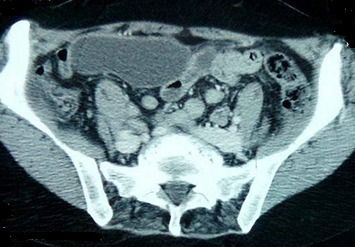
Une coupe scannographique montrant une masse kystique intrapéritonéale

**Figure 3:**
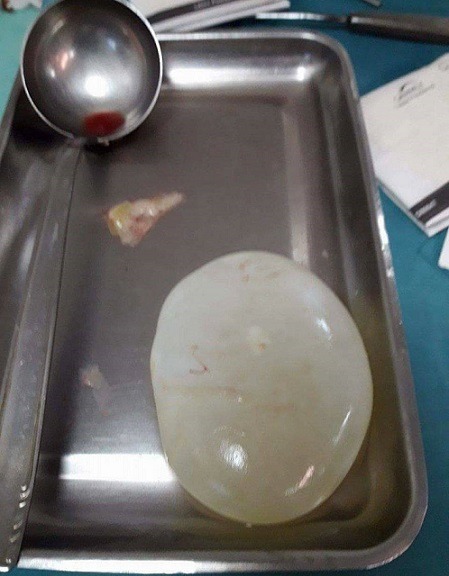
Membrane proligère intacte

**Figure 4:**
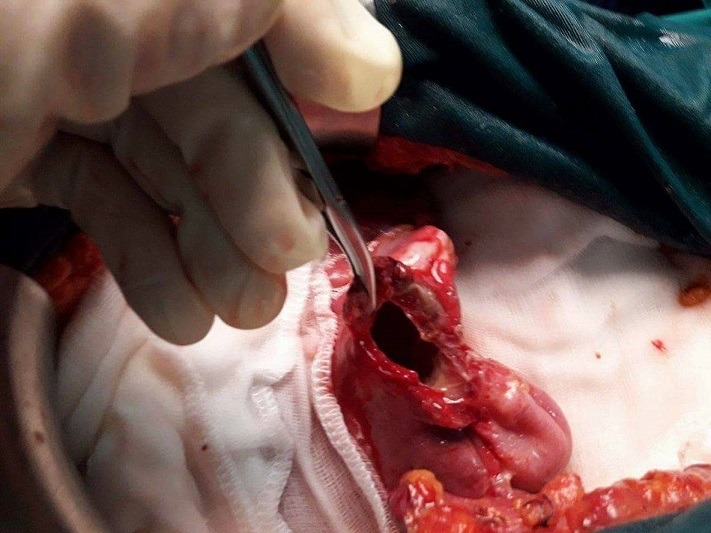
Per opératoire: résection du dôme saillant du kyste hydatique du foie

Une femme âgée de 63 ans, consultait pour douleurs de l'hypochondre droit évoluant depuis 2 mois exacerbée depuis 24 heures, sans fièvre ni ictère. L'examen trouvait une sensibilité de l'hypochondre droit. A la biologie il y avait une hyperleucocytose à 11 310 éléments/mm^3^. Sans de cholestase ni de cytolyse. L'échographie abdominale montrait un kyste du segment IV du foie associé à une lésion kystique anéchogène intrapéritonéale, mobile, uniloculaire. La tomodensitométrie abdominale montrait un kyste hydatique du segment IV affaissé et une masse kystique intrapéritonéale de 12 x 6cm sans épanchement intrapéritonéale libre (Figure 1, Figure 2). La radiographie du thorax ne montrait pas d'image de kyste pulmonaire. Le diagnostic d'une rupture intra péritonéale du kyste était évoqué et la patiente était opérer en urgence par une incision médiane. L'exploration trouvait un kyste hydatique du segment IV rompu et vidé de sa membrane proligère. Cette dernière est restée intacte en intra péritonéal. Le traitement chirurgical consistait en une résection du dôme saillant du péri kyste avec extraction de la membrane proligère et son contenu sans la rompre (Figure 3, Figure 4), associé à une toilette péritonéale au sérum salé hypertonique et drainage de la cavité péritonéale. Un traitement antiparasitaire par albendazole était prescrit. Les suites opératoires étaient simples. La patiente allait bien et n'avait pas de récidive hydatique avec un recul de 7 mois.

## Discussion

L'hydatidose est une parasitose qui sévit à l'état endémique en Tunisie [1]. Le foie est le premier organe atteint. La survenue d'une complication intéresse un tiers des kystes hydatiques du foie [2]. La rupture d'un kyste hydatique du foie (KHF) dans la cavité péritonéale est rare. Sa fréquence varie de 1 à 2%, selon les séries de la littérature [1]. Les facteurs prédictifs de cette rupture sont: le jeune âge du patient, un KHF supérieur à 10 cm, une pression intra kystique supérieure à 50 cm d'H_2_O, le siège sous capsulaire du KHF et la minceur du périkyste [1]. Cette rupture peut être favorisée par un traumatisme violent (accident de sport, accident de la voie publique) ou lors d'un effort de toux ou bien au cours de la grossesse [1,3,8]. La rupture est dite spontanée lorsqu'il n'y a pas de facteur déclenchant (70% à 80% des ruptures du KH sont spontanées) [1]. Nous rapportons une forme particulière d'une rupture intrapéritonéale spontanée d'un kyste hydatique du foie avec une membrane proligère intacte. Seuls Deux cas similaires chez des adultes et trois autres cas pédiatriques ont été rapportés (Tableau 1). L'intégrité de la membrane proligère protège contre la greffe péritonéale et la réaction allergique. Il est donc essentiel que le diagnostic soit porté avant la rupture de celle-ci. Les signes cliniques de la rupture intrapéritonéale sont habituellement bruyants (péritonite aigue généralisée-choc anaphylactique) rarement insidieux (douleurs abdominales localisées), ou totalement asymptomatiques (révélés par une hydatidose péritonéale secondairement). Les signes cliniques et biologiques de cette forme particulière de rupture avec membrane proligère intacte sont non spécifiques. C'est surtout l'imagerie qui permet d'orienter le diagnostic. L'échographie trouve un périkyste affaissé associé à une masse kystique intrapéritonéale mobile [3]. Le scanner abdominal trouve une masse kystique intrapéritonéale à paroi propre intacte associée à un périkyste affaissé avec des parois festonnées. Elle peut montrer aussi la solution de continuité au niveau du périkyste [3].

**Tableau 1:** Résumé des cas de rupture d’un KHF dans la cavité abdominale avec membrane proligère intacte dans la littérature

Auteurs [références]	Âge et sexe	Motif de consultation	Diamètre de la membrane proligère	Fistule Kysto- biliaire
S. Idrissa et al, 2017 [3]	6 ans, M	syndrome occlusif	71*48 mm	Non
Kara et al, 2008 [4]	54 ans, M	Syndrome péritonéal	100*100 mm	Oui
Acer et al., 2008 [5]	10 ans, F	Douleur abdominale localisée	104 × 82 mm	Non
Sharma et al, 2002 [6]	20 ans, M	syndrome occlusif	20*20 cm	Non
Arslan et al, 1998 [7]	12 ans, M	Douleur pelvienne et des flancs	Non précisé	Non
Notre cas	63 ans, F	Douleur hypochondre droit	120 x 60 mm	Non

L'imagerie par résonance magnétique (IRM) permet facilement de poser le diagnostic de rupture, mais aussi de surveiller l'évolution du kyste [8]. Le traitement chirurgical est urgent. Il est habituellement réalisé par laparotomie. Il se fait avant la rupture de la membrane proligère. Ce traitement chirurgical repose sur trois volets: le traitement du périkyste, l'extraction de la membrane proligère et son contenu sans la rompre et la toilette péritonéale. Le traitement du périkyste était conservateur dans tous les cas puisque le péri kyste est toujours mince est souple [3-7]. La fistule kysto-biliaire doit être systématiquement recherché et traitée. Cette fistule était présente dans un seul cas, compliquée d'une péritonite biliaire [4]. Son traitement dépend habituellement de sa taille (large, supérieure à 5mm ou mince inférieure à 5mm) et du contenu des voies biliaires (présence ou non de matériel hydatique). L'extraction du kyste hydatique intrapéritonéal doit être précautionneuse sans rompre la membrane proligère. Le lavage péritonéal au sérum physiologique additionné d'une solution scolicide dans le but d'éviter les récidives péritonéales est réalisé par tous les auteurs [3-7]. Un traitement antiparasitaire doit être administré après la chirurgie. L'albendazole est actuellement la molécule de référence [1]. Elle est prescrite à la dose de 10 à 12mg/kg/j par cure de 4 semaines, suivies de 2 semaines d'abstention, avec un maximum de 8 cures [1]. L'albendazole est contre indiqué au cours du premier trimestre de la grossesse à cause des effets embryotoxiques et tératogènes démontrés chez l'animal [8], ce qui impose une contraception adaptée chez la femme en âge de procréation. Les effets indésirables sont rares à type d'hépatite toxique (15%), de leucopénie (1,2%) et d'alopécie (2,8%) [1]. Du fait du caractère non immunisant de la maladie hydatique l'éducation thérapeutique du patient s'impose pour éviter les récidives par ré infestation, le dépistage des autres cas de maladie hydatique dans l'entourage, et de façon plus générale, les mesures d'éradication de de la maladie.

## Conclusion

La rupture intrapéritonéale du kyste hydatique du foie avec membrane proligère intacte est rare. En effet un traitement chirurgical urgent met le patient à l'abri de l'hydatidose péritonéale et de l'anaphylaxie aigue.

## Conflits d’intérêts

Les auteurs ne déclarent aucun conflit d'intérêts.
